# Reply to the “Items of Interest”

**Published:** 1901-03

**Authors:** B. F. Arrington

**Affiliations:** Goldsboro, N. C.


### Domestic Correspondence.

### REPLY TO THE "ITEMS OF INTEREST."

To the Editor:

Sir,—I must ask for limited space in your valuable journal to reply to a few points in Dr. Ottolengui's editorial reply to criticism, in the January issue of *Items of Interest.* It is with reluctance that I say anything more on the subject in question, and would prefer to remain silent, but Dr. Ottolengui in his reply says,

"Dr. Arrington entirely failed to interpret quotation, and with misinterpretation has erected an edifice full of evil."

With due respect for the doctor's view of the interpretation, I must say I interpreted the language in "Snap-Shots" which I criticised just as a large majority of fair-minded men would feel authorized to interpret, and possibly would interpret, and there was no error in regard to it. The quotation is correct, and the language is plain English and speaks for itself. There is no dodging the meaning of this language. It will admit of but one legitimate construction (interpretation), that which I gave it. This, I am sure, would be the candid opinion of all unprejudiced men in the dental profession familiar with the English language.

In writing the criticism, "What does it mean?" it was foreign to my intention to "erect an edifice full of evil," and I must say, in all frankness, if there is any such structure reared, the editor of the *Items of Interest* is the inventor and builder, and to him, single-handed, shall all the honor be accorded. He says, "The language of Dr. Arrington is neither logical nor intelligent." By his measurement, in his effort to work out of a hole, it possibly to him is so, but it was plain English frankly expressed, and conveyed a fact easily comprehended.

At the close of the last paragraph of the editor's reply he says, "They will not hesitate to act, even though some may have accused them of being hirelings with a fixed price." This is an unjust and unauthorized accusation, and has no foundation in truth to sustain it. My language used in the criticism will not admit of any such construction, unless twisted, turned, and stretched for an object. I said, "No Southern man, a member of the Southern Dental Association, unless a hireling with a fixed price (there may be such, but I am unwilling to believe it), will ever offer such an amendment under existing circumstances." Some may ask, What were the circumstances to which allusion was made? The circumstances were these: there was an arrangement and agreement for consolidation entered into at Old Point Comfort in 1897 which had not been complied with as was stipulated, except on the part of the Southern. Then, in August, 1900, in the *Items of Interest*, under the heading "Snap-Shots at the National Association," the author, R. O.,—presumably Dr. R. Ottolengui,—had the effrontery (that is what it was) to proclaim that "the idea of having subdivisions of the Association, to be known as Eastern,

Western, and Southern branches, it is well known now, was placed in the constitution as a means of inducing the Southern men to agree to union."

My language was too plain not to be understood, and should not be misconstrued to create mischief.

I make no retractions, nor do I offer any apology for language used, for I felt at the time of writing, and do now, that the circumstances stated justified the language. All I ask is that the *circumstances* be rightly considered and the language rightly interpreted.

Dr. Ottolengui, in an effort to strengthen his position, refers to and quotes his editorial in *Items of Interest,* 1897, page 800, for his ideas of what occurred at Old Point Comfort, when the occurrences were quite fresh in his memory. He says, "Before the meeting at Old Point Comfort the question of union of the Southern and American into a National Dental Society was discussed, and it was suggested that there might be tributaries in the East, South, and West. This is the plan which was submitted by the committee and was finally adopted. It seemed wise when offered, because of the fact that the love which the Southern men have for their society was such that the committee and others felt assured that any plan which would disband the Southern would fail, and that on such terms union could not be effected." And then says, "But immediately after the adoption of the committee's constitution and by-laws, union being an accomplished fact, it became evident to all who analyzed the situation that the machinery of management for the new National body was cumbersome, and if not radically altered ultimate failure must ensue." Worse and worse, but in close accord with the "*Snap-Shot*" *blunder.* The word *immediately* is expressive, but unwisely uttered. The question very naturally arises, Why did it so suddenly become evident *after the union was an accomplished fact,* "that the machinery of the management of the new National body was cumbersome, and that if not radically altered ultimate failure must ensue"? "Immediately," he says. That is a word that cannot be misconstrued. Everybody knows what the word *immediately* means. There is no twisting it for effect. The immediate evidence and the evident foreknowledge of results was a feature of wisdom somewhat surprising and unlooked for. Generally, foreknowledge is helpful and works advantageously, but in this instance it has evidenced somewhat a feature of bad faith and has worked badly, as might have been anticipated. The doctor has evidently gotten into a pretty deep hole, and there must stay until he learns to be more cautious in writing up proceedings of the National Association and ceases taking such *immediate* recognition of possible consequences.

B. F. Arrington.

Goldsboro, N. C.

